# Monthly Summary

**Published:** 1895-04

**Authors:** 


### Monthly Summary.

UTILIZING AMALGAM WASTE.—The only really economical method of utilizing amalgam waste practicable for the average dentist is to reduce it to dental alloy by remelting, and thus expelling the mercury. It can then be cast into an ingot and reduced to fillings. Prof. George T. Barker suggested it to me many years ago, and from frequent favorable notices of the process in the dental journals from time to time I presume it has proved to others, as it has to me, generally satisfactory.

If the waste is all of the same make or formula, my experience invariably has been that the product is a little better than the original. If a general mixture of all kinds, I would not advise its use in the mouth till it has been well tested out of the mouth, especially for hardness and skrinkage. If there is the least doubt of its being good it had better be thrown away.

The method is this. Place the waste in a crucible much too large, apply sufficient heat to merely fuse the mass. As the mercury is apt to be expelled somewhat explosively, cover the crucible with a slab of anything non-metallic that will resist a red heat, to prevent its contents being thrown out. When the heat reaches about 700°, the mercury begins to distil off, rising in white fumes. It is very important that these fumes be not breathed, the fire should be so arranged that they are carried off by the draft. After visible fumes cease to be given off, I hold the polished face of a cold hammer over the crucible for a moment, if any mercury is still escaping, its presence is indicated by coating the hammer with minute globules. When the mercury seems to have been completely expelled, add a little borax, increase the heat to a full bright red, and after a few moments pour into the ingot, holding back the dross with an iron rod.

To avoid disappointment, before attempting to refine amalgam waste, a little simple arithmetic may be in order. We may assume, roughly, that dental alloy prepared for amalgamation averages less than half an ounce of pure silver to each ounce of alloy; we may also assume that each ounce of waste is about half mercury. Therefore, one ounce of waste contains about one-fourth ounce of pure silver. This will hold good with most of the alloys now on the market. Gold, the only other metal of value it is likely to contain, would amount to so little in several ounces of waste that the isolation, while interesting as a chemical experiment, would cost far more than its worth. Now, pure silver is worth at the present time from sixty five to eighty five cents an ounce. We may also consider that there is always an actual loss in all refining processes; that this loss is nearly as great in refining one ounce as in refining twenty. Before beginning, we can thus proximate the value of the maximum amount of silver possible to recover. We can easily, by consulting any text book on chemistry or metallurgy, closely proximate the cost of acids, fuel, etc., required, and decide whether the probable gain will compensate for the time, the injury the acid fumes may cause to the tools in the work-room, the soiled hands, and possible injury to clothing. I have taken, so far, no account of the mercury. By placing the waste in an iron retort, with suitable connections, the mercury can be recovered. I have found it always contaminated, and not in a condition for immediate use for making dental amalgam. I so far have failed, after repeated efforts, to make it usable for that purpose at a less cost than a purchasable article. It complicates matters, and a narrow escape from accident convinced me that the attempt to save it might prove expensive economy.

In the old days—may they never return!—when we had to refine our gold and silver, and the laboratory was properly equipped for such work, and the workman in constant practice, we thought no more of it than running a plaster-cast. The method is simple enough, and may be found in many good works on metallurgy. It is interesting as a laboratory experiment, but as economy, with less than a pound or two of waste to work on, it does not pay. I have done it hundreds of times and that has been my experience.—W. H. TRUEMAN, in *Items of Interest.*

THE TONGUE IN DISEASE.—Dr. F. L. Gerald, in *Herald of Health*, says: The tongue is of great diagnostic value, and by close observation it will give us valuable aid in determining the character of disease. The tongue tells us of the condition of the blood, the conditton of the nervous system, and the functions of nutrition and excretion. As these are important things to know, we will make the tongue talk as plainly as possible. We find the expression of disease in its form, its condition of dryness or moisture, its coatings and colors. Change in form is expressive of disease. The elongated and pointed tongue indicates a condition of irritation and determination of blood to the stomach and bowels, and it is safe to give it full weight, and be careful in the administration of remedies.

As it is associated with excitation of the nerve centers, this evidence is valuable with reference to the stomach and bowels. If we observe this change of form at first, we not only anticipate unpleasant gastric irritation during the sickness, but it puts us on our guard against using anything that will irritate the stomach and bowels. The full tongue, broad and thick, is evidence of atony, want of action in the digestive tract. Then the stomach will bear cathartics in mild form without danger. The dry, pinched tongue expresses a want of functional activity in the digestive organs. It is the tongue of acute disease, and is usually associated with dryness. While it is one of the indications for food. we must be careful in its selection, giving small quantities at a time, and in a warm liquid form.

The fissured tongue in chronic disease indicates inflammatory action of the kidneys. The fissured tongue in advanced stages of acute disease refers us to lesions of the kidneys, or irritation of the nerve centers. In many cases we find a wrong in the secretion of urine. It deserves close attention and means to put the skin in better condition, and allay irritation of the nerve centers. Dryness and moisture are important evidences of the condition of the digestive organs. If the tongue is dry, we are sure the stomach and intestines can do but little digestive work. It is absurd to employ cathartics in such cases, unless the object is simply to remove irritating matter. In acute disease with dryness of tongue, when we find it becoming moist, we are confident of improvement, and it is nearly always looked on as a favorable symptom.

The thin, transparent coating of the tongue gives evidence of enfeebled digestion, frequently from intemperate eating and drinking.

A heavily-loaded tongue at the base calls attention to accumulations in the stomach. and suggests the use of an emetic The broad, pallid tongue gives evidence of the want of alkaline elements of the blood. It may be the basis of the entirety of the disease, which will fade away as soon as the proper alkali is given, or it may be but a portion of the wrong and the alkaline salt prepares the way and facilitates the action of other remedies. The deep red tongue, generally dry, indicates an acid. A dirty white or dirty gray tongue means antiseptics.

While dryness always indicates excitement of the nerve centers and calls for sedatives, too much moisture and relaxation is evidence of the opposite condition.

---

DELUSIVE PAIN.—One of the worst cases of excruiating pain I have ever known was of pure delusion. In examining a lady's mouth with a mirror she imagined that I was extracting her teeth. She broke away, left my room wringing her hands in agony, and crying aloud that I had broken her jaw and nearly killed her. It was several minutes before her husband and two lady friends could bring her back, and convince her that not even any attempt was made to work for her. Finally, when she had "come to herself," she submitted to the extraction of several teeth with unusual fortitude. Things are as we see them—to us.—*Items of Interest.*

---

MAGNESIUM HYDRATE.—For a local preparation in erosion, decalcification of enamel at the cervical border, or where the secretions are acid, as occasionally of gestation or lactation, is useful, bridging over, as it were, a period till physiological changes take place. Cases of young misses and men are numerous where an antacid is indicated. Its taste being pleasant without danger of injurious effects should place it on the front shelf as a valuable remedy to prescribe. It follows lime water, bicarbonate of soda and precipitated chalk. A teaspoonful taken into the mouth at bed-time, and rinsed around till it comes in contact with the teeth, forms and antacid coating which adheres sufficiently to protect them for several hours. In extreme cases I prescribe its use three times daily, after meals, It is known as Phillip's Milk of Magnesia.—J. R. Bell, in *Items of Interest.*

---

HE is wise who never becomes surprised or irritated by the advent of interruptions. Whatever it be—troublesome duties, illness, domestic sorrows—we all should be ready for the darkest hour. We require calmness, resignation, and a steady faith that God knows best what we need and what service we can best render. Some of our greatest achievements are in the passive side of life,—not doing, but waiting and suffering. If the fire comes, let us remember that character, like gold, needs the crucible for its finest finish. In our aim to do, let us not forget that greater purity is needed for the greater achievement, and a patient endurance of interruptions contributes to the growth of purity.—J. M, Buckley, in *Items of Interest.*

---

WHAT PART DID ANTITOXIN PLAY?—A case of recovery from tetanus, treated by antitoxin, is reported in the *British Medical Journal.* Incidentally, however, it is noted that the wounded finger was amputated and *chloral hydrate*, *bromide of potassium and morphine* freely administered.

If this is a specimen of the statistics upon which such extravagant claims are founded, it is scarcely worth while to seriously consider the subject of antitoxin therapy.—*Med. Brief.*

HYDROGEN DIOXIDE AS A BLOOD-TEST.—In addition to its medical uses, hydrogen dioxide (Marchand) can be employad to detect blood, in conjunction with freshly prepared tincture of guaiac. Although tincture of guaiac turns blue with a variety of substances, blood is not one of them. So in testing for a stain—say on clothing—moisten the spot with water, and afterwards apply a piece of white filter paper; the slightest straw-colored stain on the paper suffices. Now, add to the spot on the paper a few drops of the guaiac tincture— no coloration. Add a few drops of solution of per-oxide, when instantly the spot turns of a deep azure blue. Of course if the spot turns blue by the guaiac alone it can not be due to blood, yet it is possible blood may be present with some other substance which has that property, and hence the employment of peroxide, in that case, would be a source of fallacy. If there is no bluing by guaiac and peroxide together, then absolutely no blood is present.—Kastenbine (L. D.), in *The Odontographic Journal.*

---

BLEACHANT.—Dr. C. A Meeker, of Newark, N. J. successfully bleached a deeply discolored left superior central incisor in thirty minutes. His method, after carefully adjusting the rubber dam, is to wipe out the prepared cavity with ammonia, this to neutralize possible acidity; then with a gold probe, armed with a swab of bibulous paper saturated with pyrozone, 25 per cent., he liberally moistens the interior of the cavity and exterior surface of the tooth, evaporating the solution by repeated blasts of cold air. This method is continued till the tooth shines out again in its original color.—*Exchange.*

---

TO STOP A HEMORRHAGE FROM TOOTH EXTRACTION.—Clean the mouth of clotted blood; fill the cavity as solid as possible with cotton saturated with Monsel's solution; rinse the mouth with water, after which examine the cotton stopper or plug for a leak. If one is found, remove the cotton and repeat the operation. The mistake sometimes made is that the cotton is put in the cavity loose, to be easily forced out by the blood.—*The Traveling Quack.*

